# Supplementary figures and images for: Translocator protein (TSPO) analysis in saliva of adults with oral mucosal lesions: A preliminary study
Source: PLoS One. 2025 Sep 17;20(9):e0330065. doi: 10.1371/journal.pone.0330065 (PMC12443267; doi:10.1371/journal.pone.0330065)

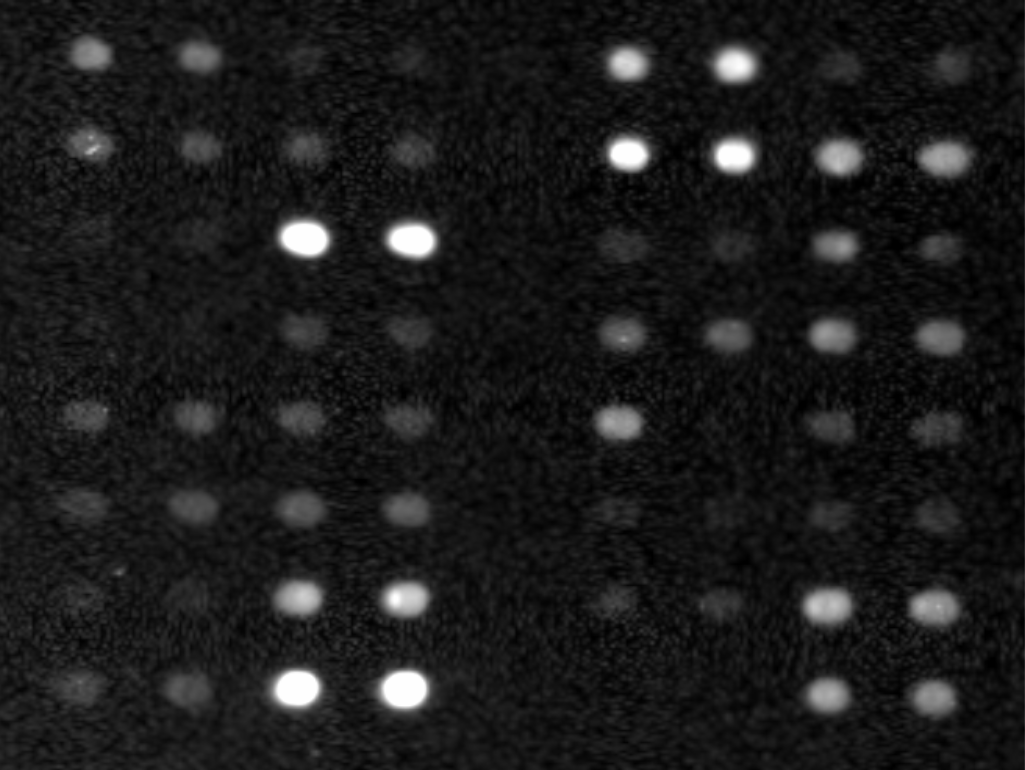

Supplement: S1 File — (TIF) [file pone.0330065.s001.tif]
