# Supplementary material for: Translocator protein (TSPO) analysis in saliva of adults with oral mucosal lesions: A preliminary study
Source: PLoS One. 2025 Sep 17;20(9):e0330065. doi: 10.1371/journal.pone.0330065 (PMC12443267; doi:10.1371/journal.pone.0330065)
Supplement: Attachment — Submitted filename: Comments (1).pdf [file pone.0330065.s004.pdf]

Comments

I would like to highlight several aspects of this study that raise questions regarding its methodology and interpretation of results.

1.Firstly, while the study presents interesting findings about the TSPO protein in saliva as a potential biomarker for oral mucosal lesions, it does not extensively address the potential confounding factors that may affect the signal intensity of TSPO. For example, how do variables such as the participants' oral hygiene, inflammatory conditions, or even dietary factors influence the protein levels in saliva? Addressing these confounders would enhance the robustness of the conclusions drawn.

2.The sample size of just 28 participants appears to be relatively small for establishing a reliable correlation between TSPO protein levels and oral lesions. Such a limited sample may not capture the variability present in a broader population. Therefore, it would be prudent for the authors to justify this sample size or discuss plans for a larger follow-up study to validate the findings presented.

3 While the study indicates that higher TSPO levels are associated with dysplasia and potentially malignant transformations, it lacks a discussion on the clinical implications of these findings. Specifically, how would the detection of TSPO protein in saliva impact current diagnostic or treatment paradigms for patients with oral lesions? Clarifying these clinical applications would enhance the relevance of the study's conclusions to practitioners in the field.

In addition to the previous points, I would also like to emphasize concerns regarding the methodological rigor of the protein detection techniques employed in this study. The authors utilized the Dot blot technique for analyzing TSPO protein levels; however, the manuscript would benefit from a more detailed description of the protocol used. For instance, how was the sensitivity and specificity of the assay validated? Moreover, were there any controls included to ensure the reliability of the results? A thorough explanation of these aspects is vital to enhance confidence in the findings.
