# Supplementary material for: Translocator protein (TSPO) analysis in saliva of adults with oral mucosal lesions: A preliminary study
Source: PLoS One. 2025 Sep 17;20(9):e0330065. doi: 10.1371/journal.pone.0330065 (PMC12443267; doi:10.1371/journal.pone.0330065)
Supplement: Attachment — Submitted filename: Response to Reviewers.docx [file pone.0330065.s006.docx]

May 16^th^, 2025

Dear Dr., Anjani Kumar Tiwari

Academic Editor

PLOS ONE

I hope this letter finds you well. As the first author of the article titled “*Translocator Protein (TSPO) Analysis in Saliva of Adults with Oral Mucosal Lesions: A Preliminary Study*”, submitted to PLOS ONE, I would like to extend my appreciation for the review provided and for the consideration of our work for publication in the journal.

The suggested changes have been reviewed in detail, and below I describe how each of these was incorporated into the revised version submitted through the system:

| **Evaluator** | **Comment or Suggestion** | **Response and/or Changes Made** |
| --- | --- | --- |
| Editor | If applicable, we recommend that you deposit your laboratory protocols in protocols.io to enhance the reproducibility of your results. Protocols.io assigns your protocol its own identifier (DOI) so that it can be cited independently in the future. | We appreciate the suggestion to publish our laboratory protocol in protocols.io. We confirmed that the protocol has been published and it has been assigned the following DOI: [**dx.doi.org/10.17504/protocols.io.kxygxqxdkv8j/v1**](https://dx.doi.org/10.17504/protocols.io.kxygxqxdkv8j/v1)  We have also included it as a reference in the revised manuscript (Reference #21). |
| Editor | Please ensure that your manuscript meets PLOS ONE's style requirements, including those for file naming. | The manuscript has been adapted to meet the journal’s style requirement as described in the instructions provided: “*Manuscript Body Formatting Guidelines*” and “*Title, Author, Affiliations Formatting Guidelines*”. |
| Editor | Please include a complete copy of PLOS’ questionnaire on inclusivity in global research in your revised manuscript. Our policy for research in this area aims to improve transparency in the reporting of research performed outside of researchers’ own country or community. The policy applies to researchers who have travelled to a different country to conduct research, research with Indigenous populations or their lands, and research on cultural artefacts. The questionnaire can also be requested at the journal’s discretion for any other submissions, even if these conditions are not met. | A complete copy of PLOS’ questionnaire on inclusivity in global research was submitted to the system as requested and added as a supplementary information in the manuscript. |
| Editor | Please note that funding information should not appear in the Acknowledgments section or other areas of your manuscript. We will only publish funding information present in the Funding Statement section of the online submission form. Please remove any funding-related text from the manuscript. | The funding information has been removed from the Acknowledgements section as requested. This information has been included in the online submission form: “*This article is a product of research funded by the Public Health Research Group from Universidad de Cartagena in Colombia. The funders had no role in study design, data collection and analysis, decision to publish, or preparation of the manuscript.*” |
| Editor | We note that you have indicated that there are restrictions to data sharing for this study. For studies involving human research participant data or other sensitive data, we encourage authors to share de-identified or anonymized data. However, when data cannot be publicly shared for ethical reasons, we allow authors to make their data sets available upon request.  Please update your Data Availability statement in the submission form accordingly. | The de-identified data set has been submitted as a Supplementary Information File #3. We confirm that the dataset has been anonymized and that the included ID information does not contain any identifiable details about the participants. Additionally, the data is being shared in accordance with local and ethical regulations. |
| Editor | PLOS ONE now requires that authors provide the original uncropped and unadjusted images underlying all blot or gel results reported in a submission’s figures or Supporting Information files. | The original and unadjusted image underlying the salivary TSPO protein dot blot from donors has been submitted as Supplementary file #1 in its original .tif format. |
| Reviewer #1 | The manuscript presents a good clinical study, when I tried to search similar articles, I found DOI: 10.1111/odi.13178 where the authors have done similar work. the authors of the current manuscript may use that information and try to draw some comparison. | We appreciate the positive feedback regarding the study. With respect to the reference of Nagler, Wizman and Gavish (2019), this key study was included as a reference in the original submission. The comparison with their work had also been previously included:  Lines 210 to 215:  “The lesions with histopathology diagnoses of dysplasia showed the highest median values of TSPO protein signal intensity in the densitometry analysis compared to other histopathological findings, which may indicate a significant presence of TSPO in dysplasia lesions. However, this difference was found not statistically significant. It has been suggested that TSPO's overexpression in malignant lesions may be associated with oral carcinogenesis (Nagler et al., 2010). **This association could be explained due to a compensatory attempt of the cell to overcome a binding capacity related malfunctioning (Nagler et al., 2019).**”  Lines 238:241:  Secondly, our comparative analyzes did not include variables related to smoking habits or cigarettes /tobacco consumption. Smoking remains the most common cause of oral cancer (P. Chaturvedi et al., 2019), **and a relationship between cigarette smoke, TSPO and oral cancer has been suggested (Nagler et al. 2010, 2019).** Thus, including the frequency of smoking habits could serve to explore the changes of TSPO presence in salivary samples in conjunction with the presence of suspicious oral mucosal lesions. |
| Reviewer #1 | language is issue so rectify the deficiencies | We appreciate the reviewer’s comment regarding language. We have carefully revised the manuscript to improve clarity and grammar throughout. The edits have been made using Track Changes, so all modifications to the language are clearly visible in the revised document. |
| Reviewer #1 | The authors obtained permission in 2018 and submitted in 2024, why so late. | We acknowledge the delay between data collection and manuscript submission. After completing the laboratory analysis in 2018, the first author began a Master's degree and subsequently a PhD program abroad, which extended the timeline for manuscript preparation. Additionally, time was required for a thorough literature review and coordination with co-authors to ensure the quality and accuracy of the final submission. We confirm that the data remain valid and the study addresses a research question that is still relevant to the field. |
| Reviewer #1 | Image resolution is issue, get these corrected | We have submitted to the journals system the images and figures with better resolution (up to 600 pixels/inch) using the .tiff format. |
| Reviewer #1 | Comparison of TSPO level with a standard is missing try to do some meaning comparison | Currently, there are no established reference values for TSPO in blood, saliva, or other body fluids. Baseline values are typically very low and can vary significantly between individuals and under different physiological stress response conditions. In this context, the relative expression levels of this protein were used to identify differential behavior between clinically well-established groups, as a first approximation of its potential role as a biomarker. Therefore, we added the following explanation to the discussion:  Lines 196 to 199:  This finding, coupled with the absence of reference values for TSPO, encourages further studies aimed at determining its baseline levels in saliva and the thresholds beyond which an increase in its concentration can be associated with the onset of dysplasia. This, along with the noninvasive nature of the test, would give this protein a promising role. |
| Reviewer #2 | In order to assess the changes in cancer cells, evaluation of biomarker is important. TSPO has been considered as biomarker for cancer. This can predict the changes at molecular level much before other modalities can predict it. The study carried out for correlation of TSPO intensity with potential malignancy could be significantly useful due to noninvasive sampling. | We thank the reviewer for this positive and encouraging comment. We agree that the non-invasive assessment of TSPO as a potential biomarker holds promise for early molecular detection of malignancy. |
| Reviewer #2 | How do we concentrate the various biomarkers taken from the saliva of the patients? | We thank the reviewer for this question. To concentrate salivary proteins, including TSPO, we applied a protein extraction and precipitation protocol using trichloroacetic acid (TCA) and CHAPS, as adapted from Jessie and Hashim <https://doi.org/10.3923/biotech.2008.686.693> This step allowed for enrichment of total proteins from saliva prior to dot blot analysis. The description of this process is presented in Figure 1, and we have published the protocol (using protocols.io) publicly:  [dx.doi.org/10.17504/protocols.io.kxygxqxdkv8j/v1](https://dx.doi.org/10.17504/protocols.io.kxygxqxdkv8j/v1)  This protocol has been included as reference #21 in the revised manuscript. |
| Reviewer #2 | Can a direct correlation be drawn between TSPO signal intensity and presence or absence of oral mucosal lesions ? If yes, then how? | While our results showed higher TSPO signal intensity in saliva samples of participants with oral mucosal lesions compared to those without, this finding should be interpreted in the context of the study’s limited sample size. As a preliminary investigation, we cannot draw a direct correlation at this stage. Rather, our findings highlight a potential association that warrants further investigation in larger, well-controlled studies. |
| Attached comments | Firstly, while the study presents interesting findings about the TSPO protein in saliva as a potential biomarker for oral mucosal lesions, it does not extensively address the potential confounding factors that may affect the signal intensity of TSPO.  For example, how do variables such as the participants' oral hygiene, inflammatory conditions, or even dietary factors influence the protein levels in saliva? Addressing these confounders would enhance the robustness of the conclusions drawn. | We agree that factors such as inflammation may influence salivary protein levels. In this preliminary study, we attempted to reduce variability by instructing all participants to refrain from eating or drinking two hours prior to sample collection and by processing all samples under standardized laboratory conditions. However, we acknowledge that we did not collect specific data on oral hygiene practices or inflammatory status, and these may represent associated factors. We have now noted this limitation in the discussion in lines 305 to 316 and emphasized the need to control for these variables in future studies.  Lines 248 to 251:  This study did not account for conditions such as oral hygiene status or subclinical oral inflammation which may affect salivary protein composition [37]. Although sample collection procedures were standardized and participants refrained from eating or drinking prior to collection, future research should control for these variables to better isolate the association between TSPO expression and oral mucosal lesions. |
| Attached comments | The sample size of just 28 participants appears to be relatively small for establishing  a reliable correlation between TSPO protein levels and oral lesions. Such a limited  sample may not capture the variability present in a broader population. Therefore, it  would be prudent for the authors to justify this sample size or discuss plans for a larger follow-up study to validate the findings presented. | We appreciate the reviewer’s concern regarding the sample size. As noted throughout the manuscript, this is a preliminary study designed to assess the feasibility of salivary TSPO protein detection in individuals with and without oral mucosal lesions. We acknowledge that the small sample size limits the generalizability of our findings and the statistical power to detect subtle associations. This limitation has been discussed in the manuscript (Lines 243:246) and further emphasized the need for larger studies to validate and expand upon these initial observations. |
| Attached comments | While the study indicates that higher TSPO levels are associated with dysplasia and potentially malignant transformations, it lacks a discussion on the clinical implications of these findings. Specifically, how would the detection of TSPO protein in saliva impact current diagnostic or treatment paradigms for patients with oral lesions? Clarifying these clinical applications would enhance the relevance of the study's conclusions to practitioners in the field. | While our study found higher TSPO levels in cases of dysplasia, this difference was not statistically significant, and we do not suggest any clinical application at this stage. However, our findings support the feasibility of salivary TSPO detection in patients with oral mucosal lesions and highlight its potential role as a non-invasive biomarker. If validated in larger studies with longitudinal follow-up, TSPO could contribute to earlier identification of lesions at risk for malignant transformation, thereby complementing clinical examination and histopathology. |
| Attached comments | In addition to the previous points, I would also like to emphasize concerns regarding the methodological rigor of the protein detection techniques employed in this study. The authors utilized the Dot blot technique for analyzing TSPO protein levels; however, the manuscript would benefit from a more detailed description of the  protocol used. For instance, how was the sensitivity and specificity of the assay validated? Moreover, were there any controls included to ensure the reliability of the  results? A thorough explanation of these aspects is vital to enhance confidence in the findings. | Regarding the absence of analysis of sensitivity and specificity in our study, currently, there are no established reference values for TSPO in blood, saliva, or other body fluids. Baseline values are typically very low and can vary significantly between individuals and under different physiological stress response conditions. In this context, the relative expression levels of this protein were used to identify differential behavior between clinically well-established groups, as a first approximation of its potential role as a biomarker. Therefore, we added the following explanation to the discussion:  Lines 196 to 199:  This finding, coupled with the absence of reference values for TSPO, encourages further studies aimed at determining its baseline levels in saliva and the thresholds beyond which an increase in its concentration can be associated with the onset of dysplasia. This, along with the noninvasive nature of the test, would give this protein a promising role. |

We appreciate the reviewers’ and editors’ constructive feedback, which has helped strengthen the clarity and quality of our manuscript

Best regards,

**Guillermo Tamayo-Cabeza, BDS, MSc**

PhD student in Dental Sciences, Research Assistant

Department of Dental Public Health and Dental Informatics

Indiana University School of Dentistry

[gtamayo@iu.edu](mailto:gtamayo@iu.edu)
